# *Cichorium intybus* L. polysaccharide improves growth performance and colonic barrier function in weaned piglets via the microbiota-HDCA-TGR5-Akt-NF-κB signaling axis: validation by FMT and in vitro models

**DOI:** 10.1186/s40104-026-01449-0

**Published:** 2026-07-06

**Authors:** Li Cao, Ge Zhang, Gang zhang, Fudong Zhang, Wenhan Li, Qian Song, Junhong He, Jinbiao Zhao, Zeyu Zhang

**Affiliations:** 1 State Key Laboratory of Animal Nutrition and Feeding, College of Animal Science and Technology, China Agricultural University, Beijing, 100193 China; 2 College of Veterinary Medicine, China Agricultural University, Beijing, 100193 China; 3 College of Animal Science and Technology, Hebei Agricultural University, Baoding, China

**Keywords:** *Cichorium intybus* L. polysaccharide, Hyodeoxycholic acid, Intestinal barrier, NF-κB pathway, TGR5-Akt axis, Weaned piglets

## Abstract

**Background:**

Weaning stress predisposes piglets to intestinal barrier disruption and gut dysbiosis, which contribute to post-weaning diarrhea and poor feed efficiency. Chicory (*Cichorium intybus* L.) polysaccharide (CLP) is a fructan-rich prebiotic candidate; however, how CLP reshapes the microbiota-metabolite network to protect the colon remains unclear.

**Methods:**

In Exp. 1, 96 weaned piglets [Duroc × (Landrace × Yorkshire), 28 days old, 8.03 ± 0.2 kg] were fed a basal diet (CON group) or a 0.5% CLP supplemented diet (CLP group). In Exp. 2, fecal microbiota from piglets were transplanted into dextran sulfate sodium (DSS)-induced mice to confirm the causal role of the CLP-remodeled microbiota. Metagenomic and untargeted metabolomic analyses were employed to identify key microbial species and functional metabolites. In Exp. 3, Caco-2 cells were treated with varying concentrations of hyodeoxycholic acid (HDCA) for 24 h to functionally validate the regulatory effects on *TGR5* and *FXR* expression levels.

**Results:**

The results showed that dietary CLP significantly decreased the feed to gain ratio, diarrhea rate and histology index (*P* < 0.05), but increased goblet cell numbers (*P* < 0.05). Metagenomic sequencing revealed that CLP significantly increased microbial α-diversity and remodeled the community structure, specifically enriching beneficial microbes, such as *Blautia* sp., *Eubacterium* sp., and *Ruminococcus* sp. To test microbiota causality, fecal microbiota from CON or CLP piglets was transplanted into antibiotic treated mice followed by DSS challenge. The CLP modified microbiota alleviates DSS induced colitis, upregulated Occludin and ZO-1 expression, and reduced colonic IL-1β and TNF-α levels. Mechanistically, the CLP remodeled microbiota promoted the accumulation of HDCA, which functioned as a signaling ligand to activate the colonic TGR5 receptor. This activation subsequently suppressed the phosphorylation of Akt (*P* < 0.05), leading to the inhibition of the NF-κB signaling pathway through the reduced phosphorylation of IκBα and the p65 subunit (*P* < 0.05), thereby effectively abrogating the inflammatory response.

**Conclusion:**

Dietary CLP supplementation mitigates weaning induced intestinal injury and inflammation by remodeling the colonic microbiota, specifically enriching HDCA-producing species. The subsequent activation of the HDCA-TGR5-Akt signaling axis inhibits the NF-κB pathway, thereby improving host immune responses and intestinal barrier function.

**Graphical Abstract:**

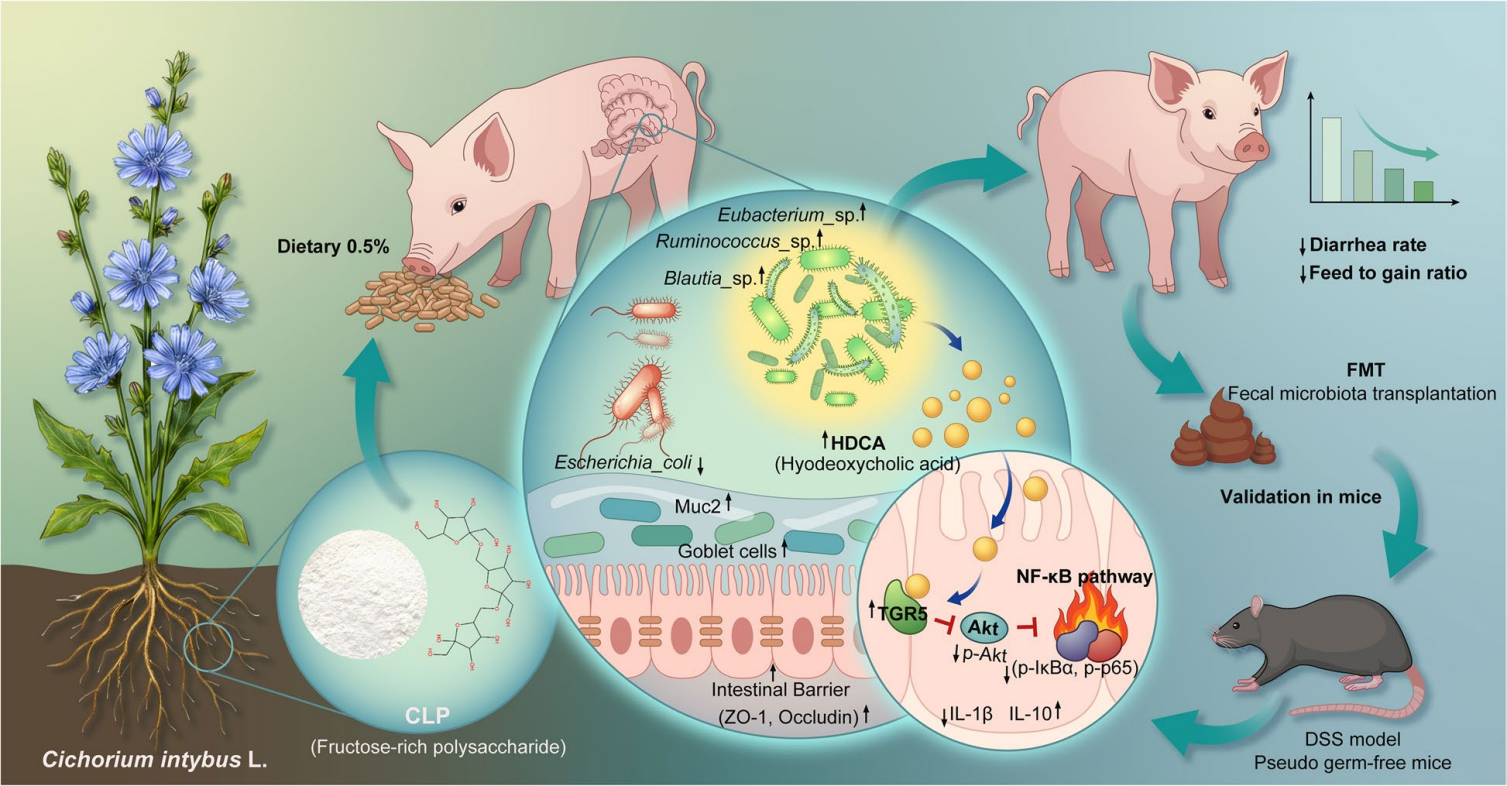

**Supplementary Information:**

The online version contains supplementary material available at 10.1186/s40104-026-01449-0.

## Background

The weaning transition represents one of the most challenging periods in the swine industry, characterized by abrupt social, environmental, and nutritional shifts. This period typically triggers weaning stress syndrome, which is manifested by severe intestinal barrier dysfunction, a sharp decline in growth performance, and high incidences of post-weaning diarrhea [1, 2]. During weaning, the immature intestinal tract of piglets is highly susceptible to dysbiosis, a state of microbial imbalance and acute inflammation, often characterized by the overactivation of pro-inflammatory signaling pathways [3, 4]. Traditionally, antibiotics have been used to mitigate these issues, but global restrictions on antibiotic growth promoters have intensified the search for sustainable, functional feed additives that can reinforce gut health through non-medicinal pathways [5].

Natural plant-derived polysaccharides have emerged as potent prebiotics due to their low toxicity and multi targeted bioactivity [6, 7]. Among these, *Cichorium intybus* L. polysaccharide (CLP) is a complex carbohydrate primarily composed of fructose units. Recent evidence suggests that CLP and inulin possess significant immunomodulatory and antioxidant properties [8–11]. In contrast to traditional nutrients, these polysaccharides reach the large intestine largely intact, where they serve as selective substrates for beneficial microbes, thereby modulating gut microbial composition and influencing host immune responses [12]. While previous studies have noted that CLP tends to improve growth performance [13, 14], the precise mechanisms, specifically how CLP remodeled microbiota coordinates with host signaling to alleviate weaning stress, remain to be fully elucidated.

The gut microbiota acts as a critical metabolic organ, converting dietary fibers into bioactive metabolites that communicate with host tissues. Among these metabolites, secondary bile acids (SBAs) have been identified as crucial signaling molecules in this crosstalk [11, 15]. Specifically, hyodeoxycholic acid (HDCA), a prominent SBA in the porcine enterohepatic circulation, has gained recognition for its ability to regulate systemic metabolism and immune responses [16, 17]. Although previous paradigms often emphasized the nuclear receptor FXR, emerging evidence suggests that these bile acids (BAs) predominantly exert their physiological effects through the G protein coupled receptor TGR5, which functions as a sophisticated anti-inflammatory switch within the colonic mucosa [18, 19]. Activation of the TGR5 signaling axis triggers downstream cascades that are essential for maintaining epithelial integrity, particularly through the inhibition of the PI3K/Akt signaling pathway to remodel the inflammatory microenvironment [20, 21]. This regulation is paramount because the NF-κB pathway remains the master signaling cascade driving the transcription of pro-inflammatory cytokines such as IL-1β and TNF-α that directly compromise intestinal barrier function [22, 23]. By suppressing Akt phosphorylation and subsequently preventing the nuclear translocation of NF-κB, TGR5 activation reinforces the expression and distribution of tight junction proteins like ZO-1 and significantly mitigates the mucosal inflammation typically induced by weaning stress [24, 25]. Consequently, the TGR5-Akt-NF-κB axis provides a more precise mechanistic framework for understanding how microbial metabolites preserve colonic homeostasis during early porcine development.

In the present study, we hypothesized that CLP improves intestinal barrier function in weaned pigs by remodeling the colonic microbiota and promoting the production of HDCA, which subsequently activates the TGR5 pathway to suppress NF-κB mediated inflammation. To test this hypothesis, we integrated a weaned piglet feeding trial with a pseudo-germ-free (PGF) mouse model for fecal microbiota transplantation (FMT) to determine whether the beneficial effects of CLP are microbiota dependent**.** Furthermore, Caco-2 cell models were employed to functionally validate the specific regulatory role of HDCA in activating the *Tgr5* expression. By combining in vivo animal models with in vitro mechanistic verification, this study aims to provide a comprehensive understanding of the interactions between dietary polysaccharides and the microbiota-metabolite-host axis, thereby providing new insights for the development of novel prebiotic interventions.

## Materials and methods

### Polysaccharide preparation

*Cichorium intybus* L. roots were obtained from Zhenhao Trading Co., Ltd. (Bozhou, China). The CLP were extracted using a hot water extraction method [26]. Briefly, the roots were washed, peeled, sliced, and dehydrated in a forced air oven at 60 °C for 10 h. The dried samples were pulverized and passed through a 1 mm mesh sieve, then stored at 4 °C and protected from light. The powdered material was mixed with distilled water at a ratio of 1:20 (w/v) and subjected to constant temperature extraction at 75 °C. After cooling to room temperature, the mixture was centrifuged at 3,300 × *g* for 15 min to remove the residue. The supernatant was then subjected to ethanol precipitation, followed by lyophilization to CLP.

The monosaccharide composition of the CLP was determined using high performance ion exchange chromatography performed at Sanshu Biotechnology Co., Ltd. (Nantong, China). Sample preparation and chromatographic separation were performed according to established protocols [27]. Briefly, the sample was hydrolyzed with 2 mol/L trifluoroacetic acid at 60 °C for 1 h in a sealed tube to release the constituent monosaccharides. After removing the excess acid by coevaporation with methanol, the residue was redissolved in deionized water and filtered through a 0.22-μm membrane. The analysis was conducted on a Thermo Scientific ICS-5000^+^ system equipped with a Dionex™ CarboPac™ PA20 column (150 mm × 3.0 mm, 10 μm). Monosaccharides were separated using a gradient elution of water, 0.1 mol/L NaOH, and 0.1 mol/L NaOH containing 0.2 mol/L NaAc at a flow rate of 0.5 mL/min. Quantitation was performed using the external standard method.

### Animals, experimental design, and management

#### Exp. 1: piglet trial

A total of 96 weaned piglets [Duroc × (Landrace × Yorkshire), 28 days old, initial body weight (BW): 8.03 ± 0.2 kg] were randomly assigned to 2 dietary treatments: a control group (CON) fed a basal diet and a CLP group fed the basal diet supplemented with 0.5% CLP. All diets were formulated to meet the nutrient requirements [28] for weaned piglets (Table 1). Each treatment consisted of 6 pens (replicates), with 8 piglets per pen (4 barrows and 4 gilts).

**Table 1:** Composition and nutrient levels of the basal diets (as-fed basis), %

Items	CON	CLP
Corn	61.56	60.66
Soybean meal	8.00	8.00
Extruded full-fat soybean	10.00	10.00
Fish meal	4.00	4.00
Whey powder	5.00	5.00
Soy protein concentrate	5.00	5.10
Soybean oil	3.00	3.30
Limestone	0.80	0.80
CaHPO_4_	1.10	1.10
NaCl	0.15	0.15
L-Lysine-HCl	0.55	0.55
DL-Methionine	0.10	0.10
L-Threonine	0.20	0.20
L-Tryptophan	0.04	0.04
CLP	-	0.50
Premix^1^	0.50	0.50
Total	100	100
Calculated nutritional levels, %		
ME, kcal/kg	3,530	3,529
CP	18.89	18.88
Total Lysine	1.47	1.48
SID Lysine	1.31	1.32
SID Methionine	0.39	0.39
SID Threonine	0.77	0.77
SID Tryptophane	0.20	0.20
Calcium	0.80	0.80
Total Phosphorus	0.63	0.62

^1^ Premix provided the following per kilogram of feed: vitamin A, 12,000 IU as vitamin A acetate; vitamin D, 2,500 IU as vitamin D_3_; vitamin E, 30 IU as DL-α-tocopheryl acetate; vitamin B_12_, 12 μg; vitamin K, 3 mg as menadione sodium bisulfate; D-pantothenic acid, 15 mg as calcium pantothenate; nicotinic acid, 40 mg; choline, 400 mg as choline chloride; Mn, 30 mg as manganese oxide; Fe, 90 mg as iron sulfate; Cu, 10 mg as copper sulfate; I, 0.35 mg as ethylenediamine dihydroiodide; and Se, 0.3 mg as sodium selenite

Piglets were housed in 1.8 m × 1.5 m × 0.8 m pens equipped with slatted plastic floors, and were provided with automatic stainless steel nipple drinkers and feeders. The pens were located in a temperature controlled facility at 28 °C with a relative humidity of 65%–75%, and the piglets were given free access to feed and water [29]. The experimental period lasted 28 d, divided into phase 1 (d 0–14) and phase 2 (d 15–28). The BW was recorded on d 0, 14, and 28 of the trial. Feed consumption was recorded daily on a pen basis by weighing the feed offered and the refusals. These records were used to calculate the average daily gain (ADG), average daily feed intake (ADFI), and feed to gain ratio (F:G) using the following formulas:

$$\mathrm{A}\mathrm{D}\mathrm{G}=\left(\mathrm{Final}\, \mathrm{B}\mathrm{W}-\mathrm{Initial}\,\mathrm{B}\mathrm{W}\right)/ \text{Number of days}$$

$$\mathrm{ADFI}=\mathrm{Total\;feed\;consumption\;per\;pen}/\left(\text{Number\;of \;days}\times \text{Number\;of\;pigs\;per pen}\right)$$

$$\mathrm{F:G}=\mathrm{A}\mathrm{D}\mathrm{F}\mathrm{I}/\mathrm{A}\mathrm{D}\mathrm{G}$$

Additionally, the diarrhea status of each piglet was monitored individually at 09:00. The diarrhea incidence was calculated according to the following formula [29]:

$$\mathrm{Diarrhea}\;\mathrm{rate}\;\left(\%\right)=\mathrm{Total}\;\mathrm{number}\;\mathrm{of}\;\mathrm{diarrhea}\;\mathrm{pigs}/\left(\mathrm{Number}\;\mathrm{of}\;\mathrm{pigs}\times\mathrm{Total}\;\mathrm{observational}\;\mathrm{days}\right)\times100\%$$

#### Exp. 2: FMT in mouse

Male C57BL/6 J mice (6 weeks old) were purchased from Sipeifu (Beijing) Biotechnology Co., Ltd. (Beijing, China). Following a one-week acclimation period, mice were subjected to an antibiotic (ABX) regimen to deplete the endogenous gut microbiota. Specifically, an antibiotic cocktail consisting of ampicillin (0.1 g/kg), metronidazole (0.1 g/kg), neomycin (0.1 g/kg), and vancomycin (0.05 g/kg) was administered daily by gavage for 7 d [30]. The resulting pseudo-GF mice were randomly assigned to two groups (*n* = 6 per group): FMT_Con (receiving feces from CON piglets) and FMT_CLP (receiving feces from CLP piglets). FMT was performed daily via oral gavage for 14 d (from d 7 to d 21) to ensure stable microbial reconstitution [30]. Subsequently, to induce experimental colitis, mice were challenged with 2.5% (w/v) dextran sulfate sodium (DSS) in their drinking water from d 14 to d 21.

For the preparation of the FMT inoculum, fecal pellets (200 mg) collected from donor piglets (CON or CLP groups) were homogenized in 1.5 mL of sterile PBS using sterile silicone beads at a frequency of 45 Hz for 1 min, followed by filtration through a 70-μm cell strainer. The antibiotic pretreated mice were then administered 200 μL of the filtered fecal homogenate daily by gavage [31].

#### Exp. 3: cell culture and drug intervention

The human intestinal epithelial cell line Caco-2 (clone C2BBe1) was obtained from the American Type Culture Collection (ATCC, Manassas, VA, USA), consistent with the experimental setting described by Han et al. [32]. Cells were maintained in Dulbecco’s Modified Eagle Medium supplemented with 10% fetal bovine serum and 1% penicillin streptomycin at 37 °C in a humidified atmosphere containing 5% CO_2_. For HDCA stimulation assay, Caco-2 cells were seeded in 6 well plates and grown to 80%−90% confluence. After 12 h of serum starvation, the cells were treated with different concentrations of 10 or 75 μmol/L HDCA (CAS No. 83-49-8; MedChemExpress LLC) for 24 h [18]. Following the incubation, cells were harvested for downstream total RNA extraction and reverse transcription quantitative PCR (RT-qPCR) analysis of *TGR5* and *TXR* mRNA expression.

### Sample collection

#### Exp. 1

From d 25 to 27, fresh fecal samples from weaned pigs were collected daily from each pen and immediately stored at −20 °C. On d 28, the fecal samples were thawed and pooled by pen (approximately 300 g of total wet weight per pen) to form a representative composite sample. Subsequently, these pooled samples were dried in a forced air oven at 65 °C for 72 h to a constant weight for the determination of apparent total tract digestibility (ATTD) of nutrients [33]. On d 28, one piglet from each pen, with a BW closest to the pen average, was selected for sampling. Selected piglets were anesthetized via intravenous injection of propofol (3 mg/kg BW), followed by euthanasia via exsanguination. Segments from the mid colon were harvested and fixed in 4% paraformaldehyde solution for histological examination [29]. The remaining tissue segments were stored at −80 °C for quantification of myeloperoxidase (MPO) and inflammatory cytokines using enzyme linked immunosorbent assay (ELISA) kits. Additionally, colonic digesta samples were collected, immediately snap frozen in liquid nitrogen, and stored for subsequent microbial and untargeted metabolomic analyses.

#### Exp. 2

On d 21, all mice were euthanized, and colons were rapidly dissected and photographed for documentation. The colon was oriented with the cecum as the proximal end and the anus as the distal end. A 1-cm segment of the distal colon was collected and fixed in 4% paraformaldehyde solution for histological analysis [34]. Colonic contents were collected and stored at −80 °C for untargeted metabolomics analysis. The remaining colonic tissues were thoroughly rinsed with ice cold PBS and stored at −80 °C for transcriptomic analysis, RT-qPCR, and Western blotting (WB) analysis.

### Hematoxylin and eosin (H&E) staining and alcian blue-periodic acid-Schiff (AB-PAS)

Colonic tissues were fixed in 4% paraformaldehyde for 24 h, followed by dehydration and embedding in paraffin wax. Paraffin Sects. (4 μm thick) were then prepared and subjected to H&E and AB-PAS staining (Servicebio, Beijing, China). Pathological scoring of tissue injury and goblet cell counting were performed in five randomly selected fields per section for each piglet and mouse, according to the criteria described. As shown in Supplementary Table S1, the histology index was calculated as the sum of the scores for epithelial damage, inflammatory infiltration, and depth of lesion [30]. Additionally, disease activity index (DAI) was assessed as follows: Starting from d 0 of DSS administration, the severity of diarrhea and fecal blood in mice were monitored daily. Combined with changes in BW, the daily DAI was calculated as the average score of these three parameters (BW loss, stool consistency, and fecal blood). The scoring was performed according to the criteria provided in Supplementary Table S2, adapted from previously methods reported by Wu et al. [35]. The dynamic changes in the DAI during the induction period were subsequently plotted as a curve to evaluate the progression of colitis.

### Determination of MPO and inflammatory cytokine in colonic tissue

Briefly, 30 mg of colonic tissue was homogenized in sterile PBS at a ratio of 1:9 (w/v) with two 3 mm grinding beads using a tissue homogenizer (70 Hz, three cycles of 60 s running and 30 s pausing). The resulting homogenate was centrifuged at 10,000 r/min for 10 min at 4 °C, and the supernatant was collected for subsequent analysis. The concentrations of MPO, interleukin-1β (IL-1β), interleukin-6 (IL-6), interleukin-10 (IL-10), and tumor necrosis factor-α (TNF-α) in the colonic tissues of piglets and mice were determined using commercial ELISA kits (Beijing Laiboterui Technology Development Co., Ltd., Beijing, China) according to the manufacturer’s instructions. Cytokine levels were determined using commercial ELISA kits and normalized to the total protein concentration of each sample, as measured by a BCA protein assay kit (Nanjing Jiancheng Bioengineering Institute, Nanjing, China). The results are expressed as ng/mg of total protein. Detailed information on the performance of these kits is provided in Table S3.

### Immunofluorescence analysis

Colonic tissues were fixed in 4% paraformaldehyde, dehydrated, embedded in paraffin, and sectioned into 4 μm thick slices [36]. For triple label four color immunofluorescence, sections were deparaffinized, rehydrated, and subjected to heat induced antigen retrieval in EDTA buffer (pH 9.0) at 100 °C for 25 min. Endogenous peroxidase activity was quenched using 3% H_2_O_2_ for 25 min, followed by blocking with 3% BSA at room temperature for 30 min to minimize non-specific binding. Multiplex staining was performed through three sequential rounds of labeling using the tyramide signal amplification (TSA) technique. Briefly, in each cycle, sections were incubated with a specific primary antibody (4 °C, 12 h), followed by a horseradish peroxidase (HRP)-labeled goat anti-mouse secondary antibody and the corresponding TSA fluorophore (iF488, iF555, or iF647). A stripping buffer was utilized between successive rounds to remove the previous antibody-HRP complex while maintaining the localized fluorescence signal. Detailed specifications of the antibodies, TSA fluorophores (including excitation/emission wavelengths), and optimized dilution rates are summarized in Supplementary Tables S4 and S5. Nuclei were counterstained with DAPI. Following the removal of autofluorescence, slides were mounted with an anti-fluorescence quenching medium. Whole slide multispectral imaging was conducted using a scanning system (Servicebio, Beijing, China), and fluorescence intensity was quantified using ImageJ software (National Institutes of Health, Bethesda, MD, USA). Briefly, regions of interest were defined by outlining the colonic epithelium along the crypt axis in five fields per section, excluding the lamina propria. After background subtraction using an unstained control, mean fluorescence intensity (MFI) was calculated per unit area (Integrated density/Area) under a standardized threshold across all groups to ensure objective comparison.

### Metagenomic sequencing of colonic digesta

Metagenomic sequencing was performed according to previous study [31]. Total genomic DNA was extracted from colonic digesta using the FastPure Stool DNA Isolation Kit (Magnetic bead) (MJYH, Shanghai, China). DNA was fragmented to approximately 350 bp using a Covaris M220 (Gene Company Limited, China). Raw paired-end reads were trimmed, and low-quality reads were removed using fastp. To avoid host interference, reads were aligned to the pig reference genome (*Sus scrofa* 11.1) using BWA, and any mapped hits associated with the host were removed. The remaining nonhost reads were processed on the Majorbio Cloud Platform for de novo assembly and functional annotation. Gene and pathway abundances were quantified and normalized to ensure comparability across samples.

### Untargeted metabolomics

Colonic contents (50 mg) from weaned piglets and mice were accurately weighed and mixed with 400 μL of ice cold 80% methanol (v/v). Samples were homogenized using a KZ-II homogenizer (Servicebio, Wuhan, China) and centrifuged at 14,000 × *g* for 15 min at 4 °C. The resulting supernatant was filtered through a 0.22-μm membrane for metabolic profiling. Quality control samples were prepared by pooling equal aliquots of all experimental samples to assess system stability. Untargeted metabolomics was conducted at Majorbio Bio-Pharm Technology Co., Ltd. (Shanghai, China) using an ExionLC AD system coupled with a TripleTOF 5600 + mass spectrometer (AB SCIEX, USA). Chromatographic separation was achieved on an ACQUITY UPLC BEH C18 column (100 mm × 2.1 mm, 1.7 μm; Waters, USA). Data acquisition was performed in both positive and negative electrospray ionization (ESI) modes, with the source temperature maintained at 550 °C. The ion spray voltage was set at 5,000 V (+) and −4,000 V (−), and the collision energy was ramped from 20 to 60 eV for MS/MS acquisition. Untargeted metabolomics was performed according to a previous study [37]. Raw data were pretreated using Progenesis QI software (Waters Corporation, Milford, USA). Only metabolites with a coefficient of variation less than 30% in QC samples were retained as reproducible measurements to evaluate system stability and repeatability. Identification was performed by matching tandem mass spectra and mass to charge ratios values against public and proprietary databases, corresponding to metabolomics standards initiative (MSI) level 2.

### RNA sequencing analysis

RNA-seq was carried out following a previous study [38]. Briefly, total RNA was extracted from tissues in triplicate (*n* = 6). RNA-seq libraries were sequenced on the Illumina platform (NovaSeq 6000) with 2 × 150 bp read length. Raw reads were quality control using fastp and aligned to the *Sus scrofa* reference genome using HISAT2. The expression level of each transcript was calculated using the TPM (Transcripts Per Million) method, and transcript abundance was quantified using the RSEM software tool. Differential expression analysis (DEGs) was performed using DESeq2, with significance defined as an adjusted *P*-value < 0.05 and |log_2_FC| > 1.

### RT-qPCR

Total RNA was extracted using Total RNA Extractor (Trizol, Sangon Biotech, China; Cat. No. B511311) according to the manufacturer’s instructions. Reverse transcription was performed in duplicate using Maxima Reverse Transcriptase (Thermo Scientific, USA; Cat. No. EP0743), and RT-qPCR was conducted using SGExcel FastSYBR Mixture (Sangon Biotech, China; Cat. No. B532955) on a Light Cycler^®^ 480 II System (Roche, Rotkreuz, Switzerland). Primers for RT-qPCR were synthesized by Tsingke Biotechnology Co., Ltd. (Beijing, China), and shown in Table S6. The relative gene expressions were calculated using the 2^−ΔΔCt^ method using *GAPDH* as the reference gene.

### Western blot analysis

Colonic tissue proteins were extracted using RIPA lysis buffer supplemented with phenylmethylsulfonyl fluoride to prevent proteolytic degradation. The total protein concentration was quantified through the BCA method. Subsequently, proteins were separated by sodium dodecyl sulfate polyacrylamide gel electrophoresis according to molecular weight and then transferred onto polyvinylidene difluoride membranes. The membranes were blocked in TBST containing skimmed milk at 25 °C for 1 h and incubated overnight at 4 °C with the appropriate primary antibodies. After several TBST washes, the membranes were exposed to HRP-conjugated secondary antibodies for 1 h. Protein bands were visualized using a Tanon 5200 Chemiluminescent Imaging System (Shanghai, China). All chemical reagents for Western blotting were obtained from Beyotime Biotechnology (Shanghai, China). Densitometric quantification of protein bands was carried out using ImageJ 6.0 software. Detailed information regarding antibodies, including source species, vendor, and dilution ratios, is provided in Table S7.

### Molecular docking

Molecular docking was performed to investigate the binding interactions between HDCA and human TGR5 or FXR, following a previous study [39]. The protein structures of human TGR5 (UniProt ID: Q8TDU6) and FXR (UniProt ID: Q96RI1), and the 3D structure of HDCA (PubChem CID: 5283820) were retrieved from the AlphaFold Protein Structure Database and PubChem, respectively. In the docking simulations, TGR5 or FXR was designated as the receptor and HDCA as the ligand. The receptor was prepared by removing water molecules and heteroatoms, followed by the addition of polar hydrogen atoms using AutoDockTools. The ligand structure was subjected to energy minimization prior to docking to ensure an optimized conformation. Docking simulations were executed using AutoDock Vina (v.1.2.0), with the search grid centered on the canonical pocket for ligand binding and an exhaustiveness parameter set to 32 to ensure a comprehensive search of the conformational space [40]. The optimal binding pose was identified based on the lowest binding affinity (kcal/mol) and visualized using PyMOL (v.4.3.0).

### Statistical analysis

All data were analyzed using SPSS (version 26.0). Normality and homogeneity of variance were assessed using the Shapiro–Wilk and Levene’s tests, respectively. For two-group comparisons, an unpaired Student’s *t*-test was used for parametric data and the Mann–Whitney U test for non-parametric data. Diarrhea incidence was analyzed using the Chi-square test. For metagenomics, α-diversity was compared using the Wilcoxon rank-sum test, β-diversity was evaluated by PCoA based on Bray–Curtis distances and validated by ANOSIM. To control for multiple testing, *P* values were adjusted using the Benjamini–Hochberg method. Differential taxa were identified by LEfSe (LDA > 3.0). Statistical significance was defined as *P* < 0.05.

## Results

### Monosaccharide composition of CLP

**Fig. 1:**
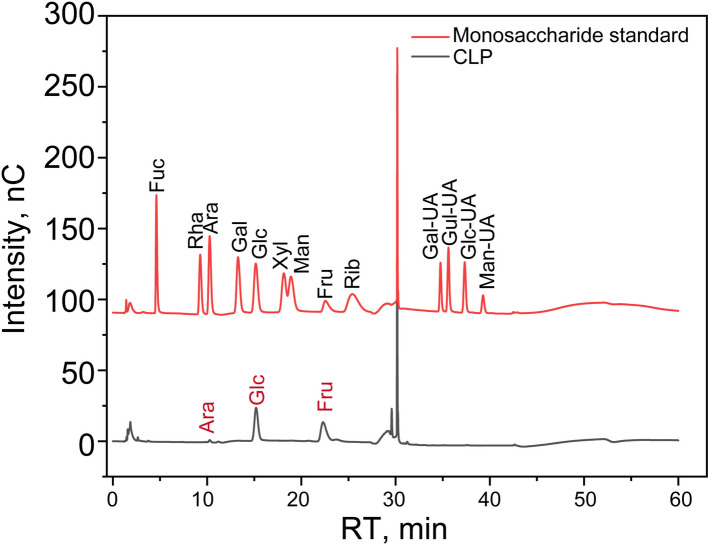
Monosaccharide composition analysis of CLP. The sample was hydrolyzed with 2 mol/L TFA at 60 °C for 1 h. Monosaccharides were identified by comparison with standards and quantified by the external standard method. Fuc, fucose; Rha, rhamnose; Ara, arabinose; Gal, galactose; Glc, glucose; Xyl, xylose; Man, mannose; Fru, fructose; Rib, ribose; Gal-UA, galacturonic acid; Gul-UA, guluronic acid; Glc-UA, glucuronic acid; Man-UA, mannuronic acid

The monosaccharide composition of CLP is presented in Fig. 1 and Table 2. The results indicated that CLP was primarily composed of fructose (81.34%) and glucose (17.96%), with a minor amount of arabinose (0.70%). CLP was rich in fructose with glucose as a minor component, consistent with an inulin-type fructan rich in fructose.

**Table 2:** Monosaccharide composition of CLP

Item, %	Arabinose	Glucose	Fructose	Total
CLP	0.70	17.96	81.34	100.00

### Effects of CLP supplementation on growth performance and nutrient digestibility of weaned piglets

Growth performance is shown in Table 3. Initial BW, BW at d 14, and final BW did not differ between groups (*P* > 0.05). During d 14–28, piglets fed CLP showed a lower feed to gain ratio (*P* < 0.05). Across the entire 28 d trial, CLP improved feed efficiency (F:G; *P* = 0.01) and tended to increase ADG (*P* = 0.09), without affecting ADFI (*P* > 0.05).

**Table 3:** Effects of CLP supplementation on growth performance of weaned piglets (*n* = 6)

Items	CON	CLP	SEM	*P* value
Initial BW, kg	8.03	8.03	0.25	1.00
Medial BW, kg	12.51	12.98	0.32	0.49
Final BW, kg	18.67	19.60	0.44	0.31
Growth performance				
d 0–14				
ADG, g/d	320	353	9.89	0.09
ADFI, g/d	476	495	13.92	0.52
F:G	1.49	1.40	0.03	0.16
d 14–28				
ADG, g/d	440	473	11.09	0.14
ADFI, g/d	714	720	15.21	0.87
F:G	1.63	1.52	0.02	0.01
d 0–28				
ADG, g/d	380	413	9.78	0.09
ADFI, g/d	595	607	13.68	0.68
F:G	1.57	1.47	0.02	0.01

*CON *Basal diet, *CLP *Basal diet with 0.5% CLP, *ADG *Average daily gain, *ADFI *Average daily feed intake, *F:G *Geed to gain ratio

Apparent total tract digestibility of gross energy (GE), dry matter (DM), organic matter (OM), crude protein (CP), neutral detergent fiber (NDF), and acid detergent fiber (ADF) did not differ between groups (Table 4; *P* > 0.05).

**Table 4:** Effects of CLP supplementation on nutrient digestibility of weaned piglets (*n* = 6)

Items, %	CON	CLP	SEM	*P* value
GE	82.61	84.18	0.71	0.29
DM	82.72	84.40	0.67	0.22
OM	86.26	87.59	0.56	0.26
CP	75.73	78.45	1.06	0.22
NDF	50.03	53.71	1.64	0.28
ADF	41.48	43.22	1.98	0.68

*CON *Basal diet, *CLP *Basal diet with 0.5% CLP, *GE *Gross energy, *DM *Dry matter, *OM *Organic matter, *CP *Crude protein, *NDF *Neutral detergent fiber, *ADF *Acid detergent fiber

### Effects of CLP on diarrhea rate, colonic morphology and inflammatory cytokines in weaned piglets

**Fig. 2:**
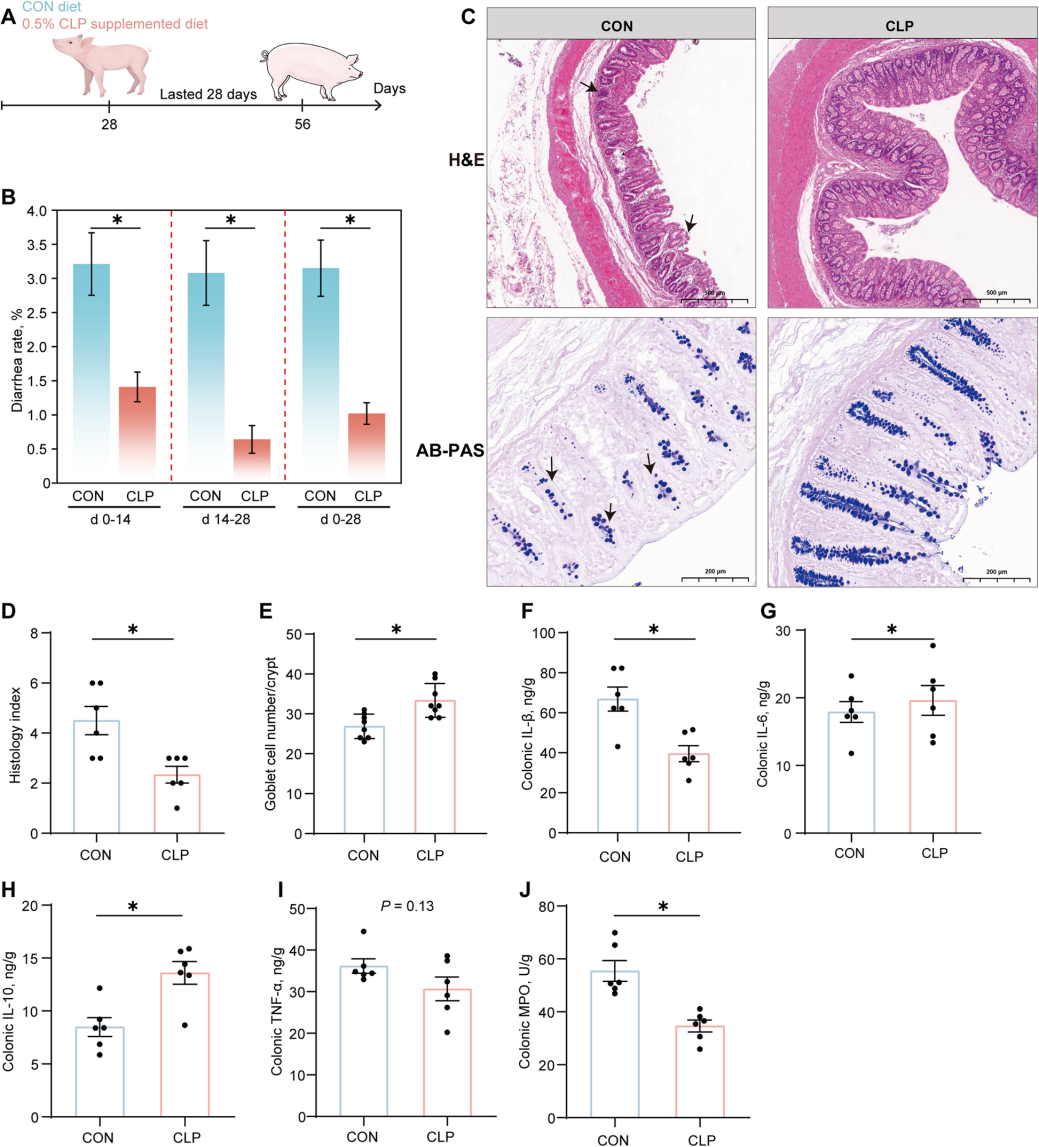
Effects of CLP supplementation on diarrhea rate, colonic morphology and inflammatory cytokines in weaned piglets. **A** Experimental schematic. **B** Diarrhea rate (%) of piglets during the phase 1 (d 0–14), phase 2 (d 14–28), and overall (d 0–28) experimental periods. **C** Representative images of H&E staining (scale bar = 500 μm) and AB-PAS staining (scale bar = 200 μm) of colonic tissues. **D** and **E** Statistical analysis of the histology index (**D**) and goblet cell number per crypt (**E**) based on colonic sections. **F–J** Concentrations of pro-inflammatory cytokines IL-1β (**F**), IL-6 (**G**), TNF-α (**I**), anti-inflammatory cytokine IL-10 (**H**), and MPO activity (**J**) in the colonic tissues. Data are presented as mean ± SEM (*n* = 6). ^*^P < 0.05. CON, basal diet. CLP, basal diet with 0.5% CLP group

As shown in Fig. 2, dietary CLP reduced diarrhea incidence during d 0–14, d 14–28, and d 0–28 (*P* < 0.05). Histological analysis revealed improved mucosal architecture and increased goblet cell numbers in the CLP group, accompanied by a lower histology index (*P* < 0.05).

In colonic tissues, CLP reduced IL-1β and MPO activity (*P* < 0.05) and increased IL-10 (*P* < 0.05). IL-6 was increased in the CLP group (*P* < 0.05), which may reflect a context-dependent response in mucosal repair and warrants further investigation. TNF-α did not differ significantly (*P* > 0.05).

### CLP reshaped the colonic microbiota in weaned piglets

**Fig. 3:**
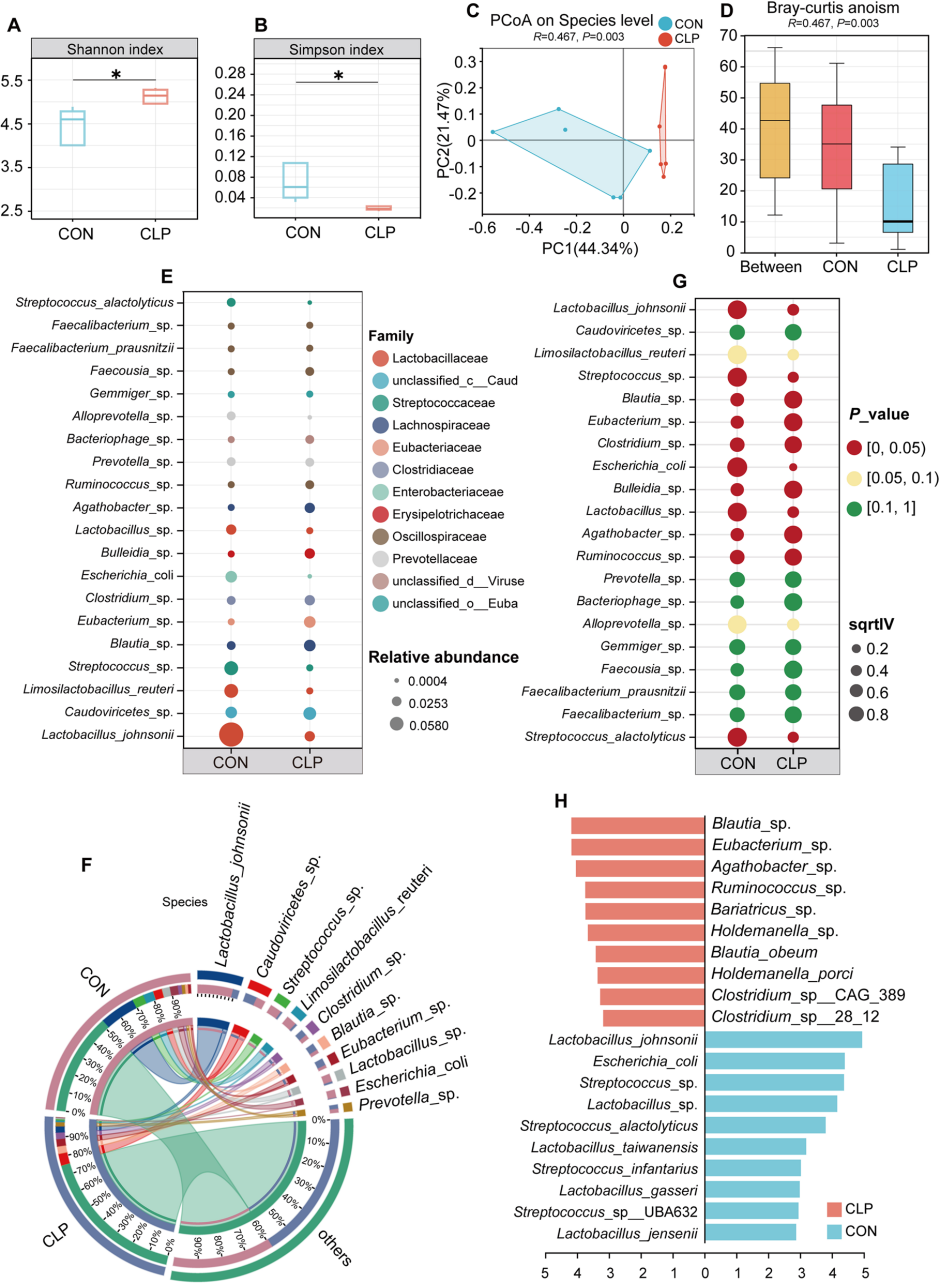
Effects of CLP supplementation on colonic microbiota in weaned piglets. **A** and **B** Microbial community α-diversity was assessed using the Shannon index and Simpson index based on species-level profiles. **C** PCoA and **D** Bray–Curtis Anosim analysis illustrating β-diversity of colonic microbiome. **E** Bubble plot showing species annotation and relative abundance at the species-level (bubble size) across different treatment groups, with phylogenetic family level information indicated by bubble color. **F** Circos plot: The left half indicates the grouping information (CON and CLP), while the right half depicts the dominant species and their relative abundances; wider segments represent higher abundance. **G** Biomarker selection via indicator analysis: the sqrtIV value represents the square root of the indicator value, where higher values reflect stronger potential as biomarkers for a given treatment group. **H** LEfSe analysis identifying differentially enriched microbial taxa between the CON and CLP groups (LDA > 3.0). ^*^P < 0.05. CON, basal diet. CLP, basal diet with 0.5% CLP group

Shotgun metagenomics revealed higher α-diversity in the CLP group (Shannon index increased; Simpson dominance index decreased; *P* < 0.05; Fig. 3A and B) and a distinct community structure (PCoA; ANOSIM *R* = 0.467, *P* = 0.003; Fig. 3C and D). Differential abundance analyses (indicator analysis and LEfSe) identified enrichment of several fiber-associated taxa (e.g., *Blautia*_sp*.*, *Eubacterium*_sp*., Agathobacter*_sp*.*, *Ruminococcus*_sp*.*, and *Clostridium*_sp*.*) and depletion of *Escherichia coli* in the CLP group (Fig. 3G and H).

### FMT from CLP piglets alleviated DSS-induced colitis in mice

**Fig. 4:**
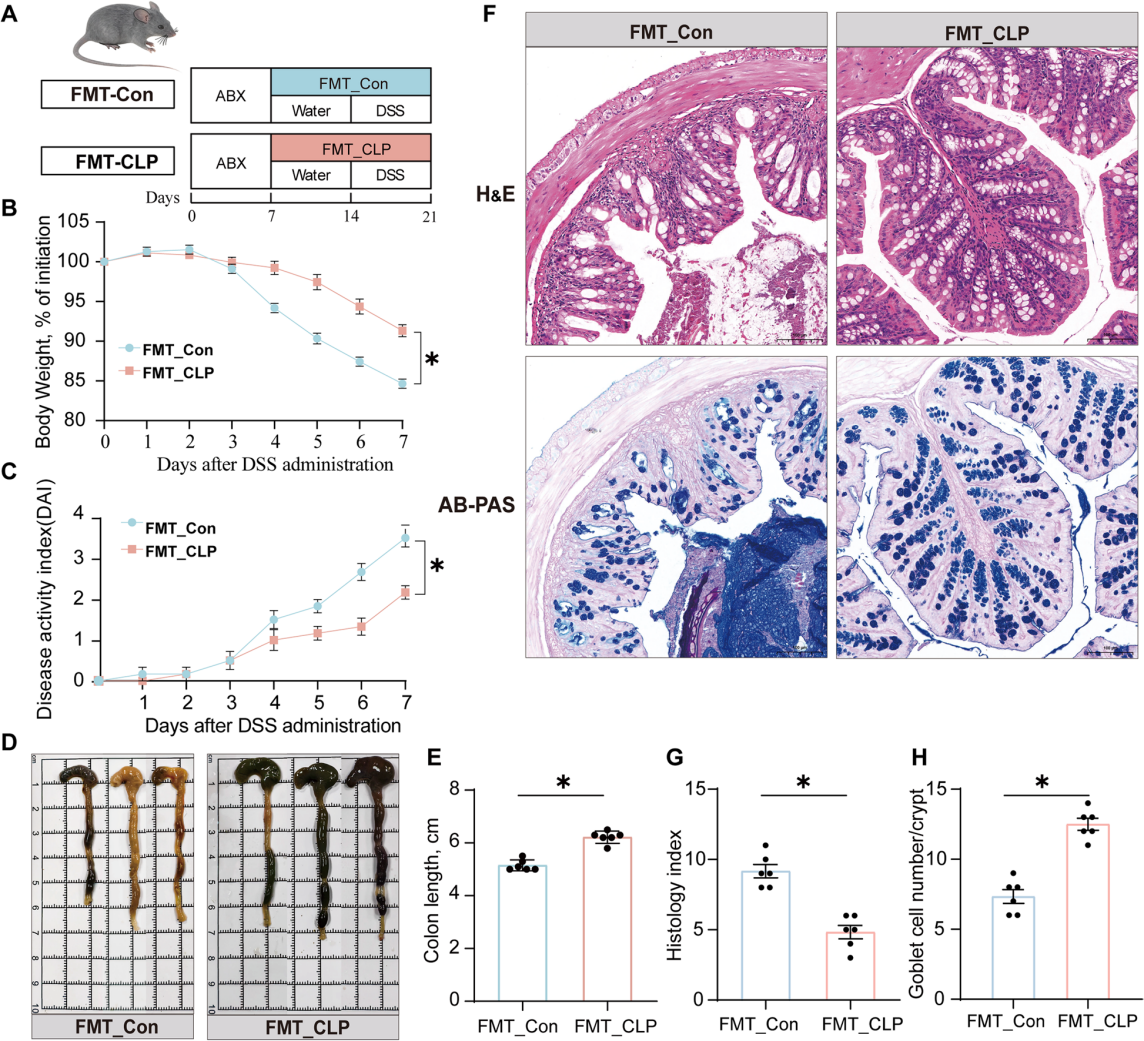
Effects of FMT on clinical symptoms, colonic morphology and histopathology in DSS-induced mice. **A** Experimental schematic. **B** and **C** Clinical indicators of colitis severity, including daily body weight changes (**B**) and disease activity index (DAI) scores (**C**) over 7 days of DSS administration. **D** and **E** Representative photographs of colons (**D**) and statistical analysis of colon length (**E**) among the groups. **F** Representative histological images of the distal colon (scale bar = 100 μm): H&E staining (top), AB-PAS staining (middle). **G** and **H** Statistical quantification of the histology index (**G**) and goblet cell number per crypt (**H**) based on histological sections. Data are presented as mean ± SEM (*n* = 6). ^*^P < 0.05 indicates a significant difference between the FMT_Con and FMT_CLP groups. FMT_Con, mice receiving fecal microbiota from the CON piglets. FMT_CLP, mice receiving fecal microbiota from CLP piglets

**Fig. 5:**
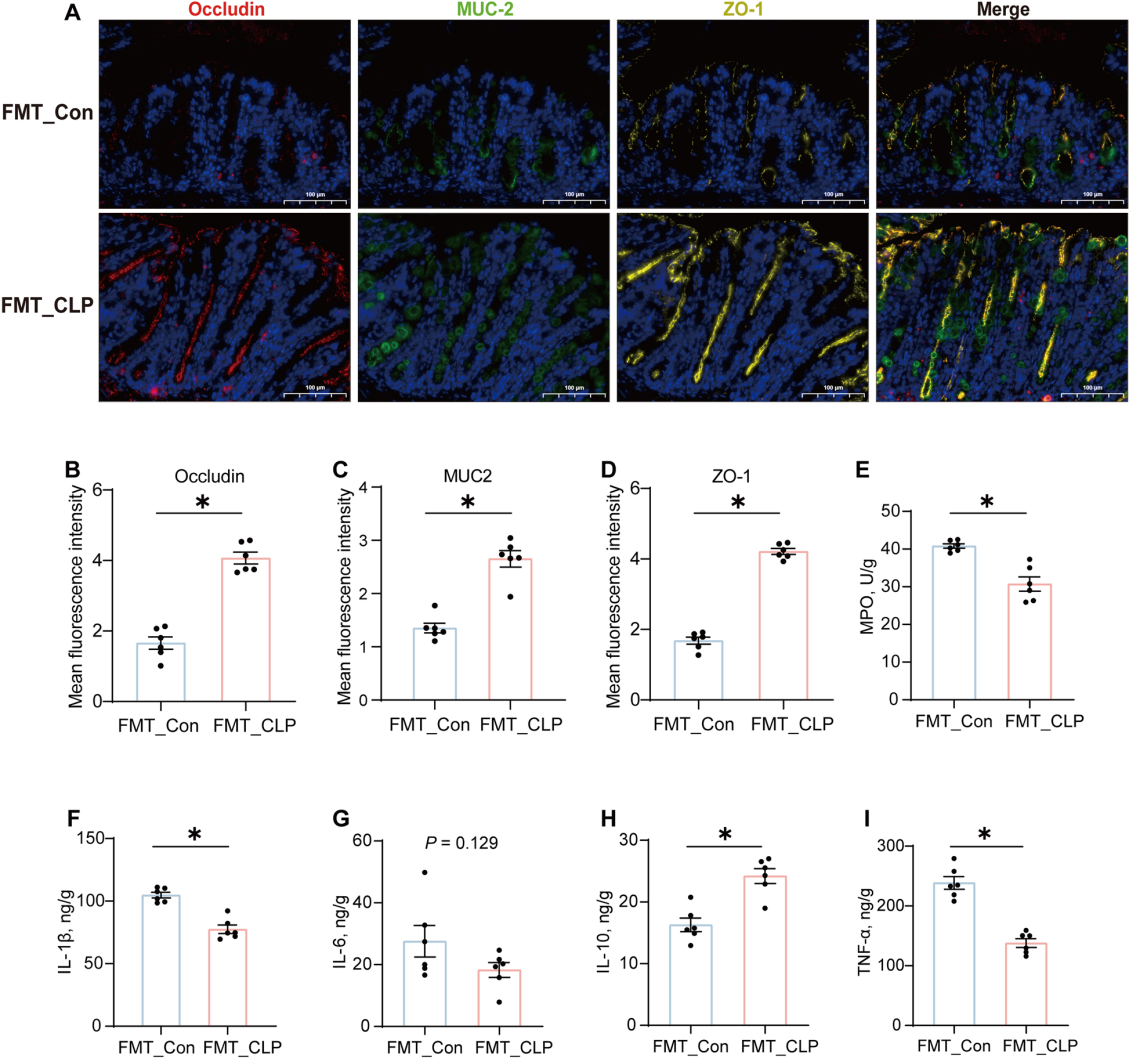
Effects of FMT on intestinal barrier and inflammatory cytokines in DSS-induced mice. **A** Immunofluorescence staining for tight junction proteins (Occludin and ZO-1) and mucin (MUC2). **B**–**D** Mean fluorescence intensity analysis for Occludin (**B**), MUC2 (**C**), and ZO-1 (**D**) in colonic tissues. **E**–**I** Concentrations of colonic MPO activity (**E**) and inflammatory cytokines, including pro-inflammatory IL-1β (**F**), IL-6 (**G**), TNF-α (**H**), and anti-inflammatory IL-10 (**I**). Data are presented as mean ± SEM (*n* = 6). ^*^P < 0.05 indicates a significant difference between the FMT_Con and FMT_CLP groups. FMT_Con, mice receiving fecal microbiota from the CON piglets. FMT_CLP, mice receiving fecal microbiota from CLP piglets

To test microbiota causality, fecal microbiota from CON or CLP piglets was transplanted into antibiotic-treated mice prior to DSS challenge (Fig. 4A). Compared with FMT_Con, FMT_CLP attenuated DSS-induced BW loss and reduced DAI (*P* < 0.05; Fig. 4B and C). Colon shortening was also alleviated (*P* < 0.05; Fig. 4D and E). Histology showed reduced tissue injury and increased goblet cells in FMT_CLP group (*P* < 0.05; Fig. 4F–H). Barrier markers (Occludin, ZO-1, and MUC2) were increased in FMT_CLP group (Fig. 5A–D). MPO activity and pro-inflammatory cytokines (IL-1β and TNF-α) were decreased, whereas IL-10 increased (*P* < 0.05; Fig. 5E–I).

### CLP-associated metabolite features in piglets and recipient mice

**Fig. 6:**
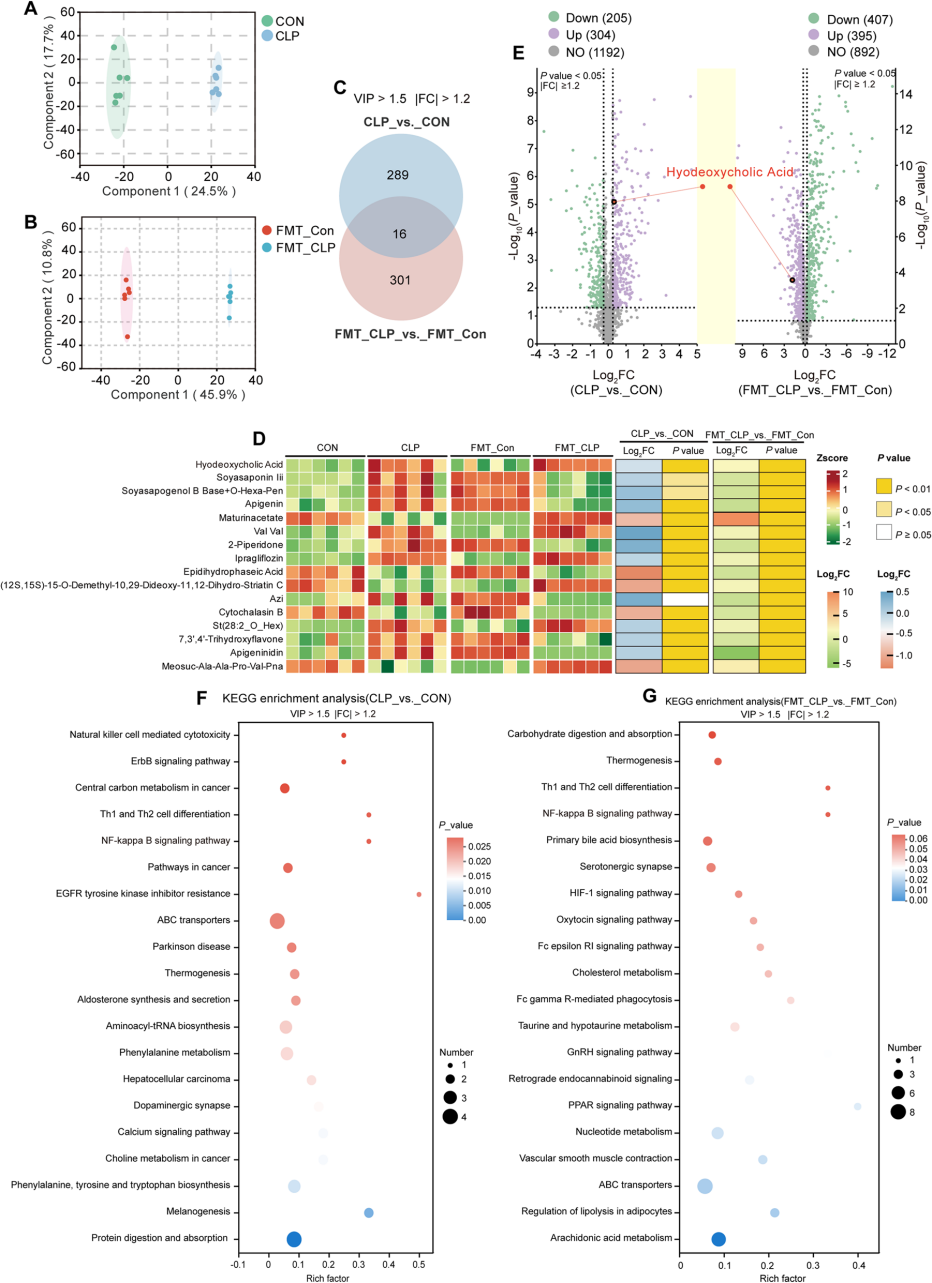
Effects of CLP on colonic metabolites in weaned piglets and FMT-treated mice. **A** and **B** OPLS-DA score plots illustrating distinct metabolic separation between the CON and CLP groups in weaned piglets (**A**) and between the FMT_Con and FMT_CLP groups in DSS-induced mice (**B**). **C** Venn diagram showing the intersection of differential metabolites between the piglet and mouse trials. **D** Heatmap illustrating the relative abundance of top differential metabolites across the four experimental groups. **E** Dual volcano plots visualizing the distribution of differential metabolites. **F** and **G** KEGG enrichment analysis based on the differential metabolites in piglets (**F**) and FMT mice (**G**). Differential metabolites were screened using the criteria: VIP > 1.5 and FC > 1.2. CON, basal diet. CLP, basal diet with 0.5% CLP group, FMT_Con, mice receiving fecal microbiota from the CON piglets. FMT_CLP, mice receiving fecal microbiota from CLP piglets

Untargeted metabolomics showed distinct clustering between groups in piglets and recipient mice (Fig. 6A and B). Sixteen differential metabolites overlapped between the piglet and mouse datasets (Fig. 6C and D). Among them, HDCA was consistently elevated in the CLP piglets and in FMT_CLP recipient mice. Given the inherent uncertainty of untargeted annotations, key bile acid features (including HDCA) were prioritized for biological interpretation. KEGG enrichment indicated that differential metabolites were mainly involved in digestion/absorption and bile acid related pathways (Fig. 6F and G).

### FMT altered colonic transcriptome and was accompanied by TGR5/Akt induction and NF-κB suppression

**Fig. 7:**
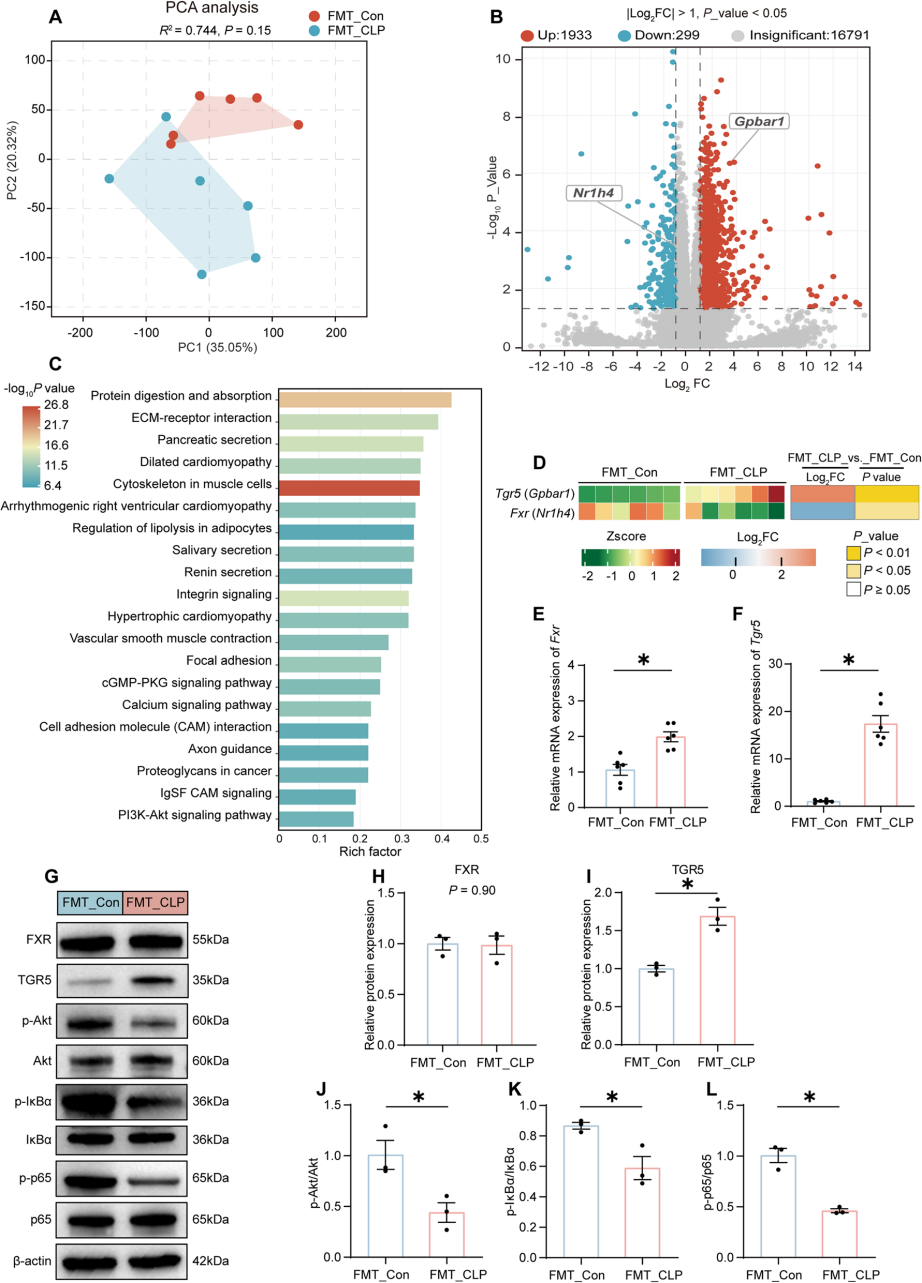
Effects of FMT on transcriptome and signaling pathways in DSS-induced colitis mice. **A** PLS-DA score plot illustrating the distinct separation of colonic gene expression profiles between the FMT_Con and FMT_CLP groups. **B** Volcano plot visualizing DEGs in colonic tissues. **C** KEGG enrichment analysis of DEGs showing the top enriched biological pathways. **D** Heatmap showing the relative expression (Z-score) and log_2_ FC of *Tgr5* and *Fxr* between groups. **E** and **F** qPCR validation of the relative mRNA expression levels of *Fxr* (**E**) and *Tgr5* (**F**) in colonic tissues (*n* = 6). **G** Representative Western blot bands for FXR (55 kDa), TGR5 (28 kDa), p-Akt (60 kDa), Akt (60 kDa), p-IκBα (36 kDa), IκBα (36 kDa), p-p65 (65 kDa), p65 (65 kDa), and β-actin (42 kDa) in colonic tissues. **H** and **I** Statistical quantification of the relative protein expression levels of FXR (**H**), TGR5 (**I**) (*n* = 3). **J**–**L** Quantification of the p-Akt/Akt, p-IκBα/IκBα and p-p65/p65 ratio. Data are presented as mean ± SEM. ^*^P < 0.05 indicates a significant difference between the FMT_Con and FMT_CLP groups. FMT_Con, mice receiving fecal microbiota from the CON piglets. FMT_CLP, mice receiving fecal microbiota from CLP piglets

**Fig. 8:**
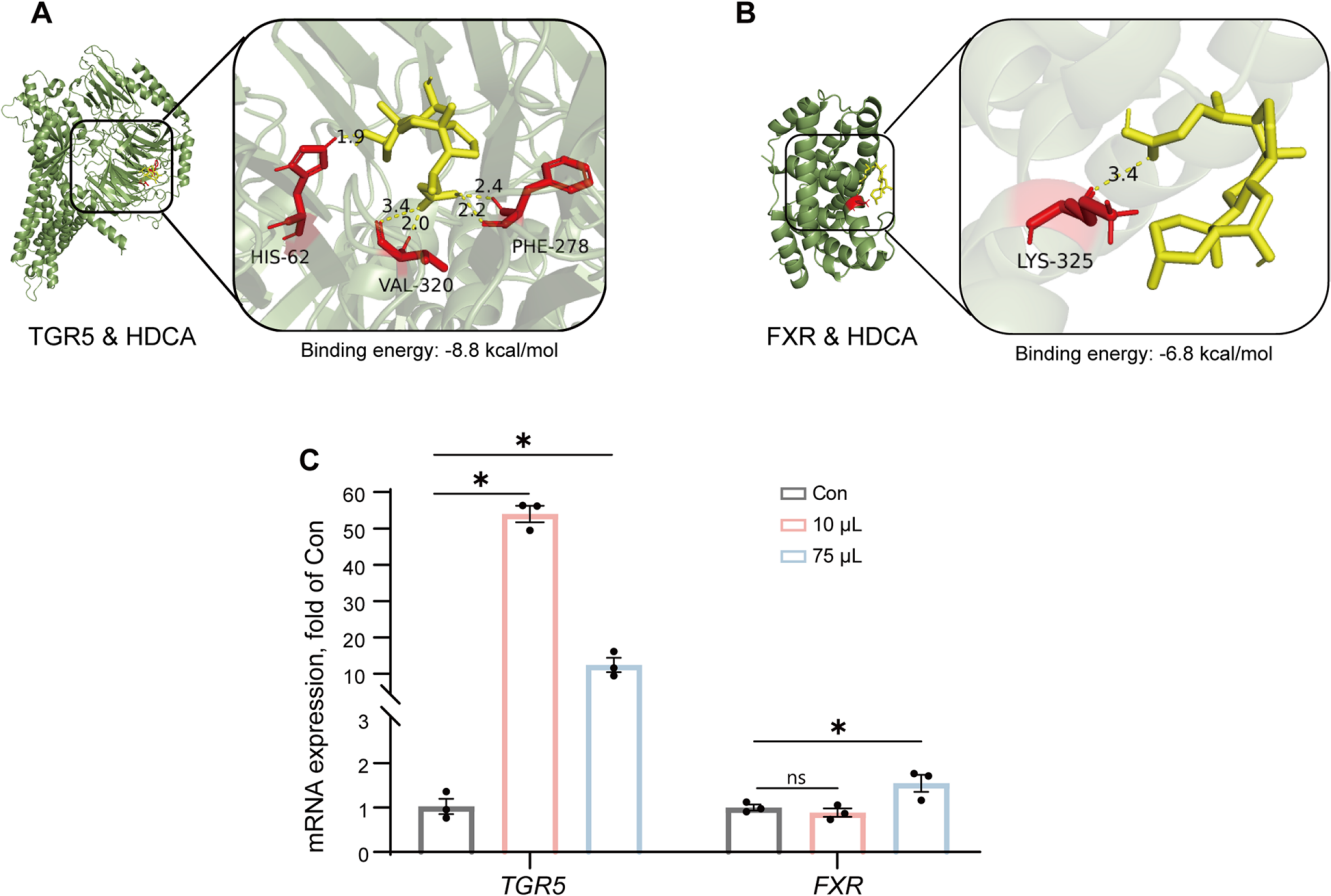
Molecular docking and in vitro validation of the interaction between HDCA and the TGR5/FXR signaling axes **A **and** B** Molecular docking analysis showing the binding modes and predicted affinities of HDCA with TGR5 or FXR. **C** Quantitative validation of the HDCA-mediated activation of the *TGR5* and *FXR* in Caco-2 cells (*n* = 3). Data are presented as mean ± SEM. ^*^P < 0.05 indicates a significant difference between the Con and HDCA groups

Transcriptomic profiling showed distinct separation between FMT_Con and FMT_CLP groups (Fig. 7A). A total of 2,232 DEGs were identified (1,933 upregulated and 299 downregulated; Fig. 7B). KEGG analysis indicated enrichment of PI3K-Akt signaling pathway (Fig. 7C). *Tgr5* (*Gpbar1*) (*P* < 0.05) and *Fxr* (*Nr1h4*) expression increased in FMT_CLP mice, which was validated by RT-qPCR and Western blot (Fig. 7D–F). In parallel, phosphorylation of Akt, IκBα and p65 decreased in FMT_CLP mice, suggesting reduced Akt and NF-κB activation (*P* < 0.05; Fig. 7G–L). Molecular docking revealed that HDCA exhibits a stronger binding affinity for TGR5 than FXR (−8.8 kcal/mol vs. −6.8 kcal/mol; Fig. 8A and B). Consistently, treatment with different concentrations of HDCA for 24 h significantly upregulated the mRNA levels of *TGR5* and *FXR* in Caco-2 cells in a dose dependent manner, with *TGR5* showing higher relative expression levels (*P* < 0.05; Fig. 8C).

## Discussions

### CLP improved feed efficiency and colonic barrier function without altering nutrient digestibility

Weaning stress is frequently accompanied by diarrhea and impaired growth performance, largely driven by intestinal barrier disruption and mucosal inflammation [1]. In the present study, dietary supplementation with 0.5% CLP improved feed efficiency and reduced diarrhea incidence in weaned piglets. Notably, these benefits occurred without significant changes in apparent total tract digestibility of nutrients, suggesting that the positive effects of CLP are likely associated with the modulation of the gut ecosystem, which alleviates inflammation-related metabolic burden. This observation is consistent with previous reports indicating that certain prebiotic fibers can improve clinical outcomes and gut integrity independently of changes in nutrient digestibility [41–44]. For instance, β-glucan has been shown to improve growth performance and intestinal epithelial functions in *Escherichia coli* challenged piglets through immunomodulatory signaling rather than caloric enhancement [45]. Similarly, galacto-oligosaccharides were found to improve immune responses and antioxidant capacity in weaned piglets, acting through microbiota-mediated mechanisms rather than as direct nutrient sources [41]. In contrast, a high level of indigestible dietary protein has been reported to impair growth and exacerbate health status in weaned piglets by fostering a detrimental proteolytic environment and promoting the proliferation of pathogens such as *Salmonella* [46]. This divergence suggests that the physiological impact of the indigestible dietary fraction depends heavily on its fermentation characteristics. In summary, our results highlight that CLP functions as a potent bioactive modulator that stabilizes colonic homeostasis independently of changes in nutrient digestibility. Instead, the beneficial effects of CLP are primarily associated with its capacity to remodel the colonic microbial community, which appears to be a critical factor in maintaining intestinal integrity and physiological homeostasis.

### CLP remodeled the colonic microbiota and was associated with HDCA enrichment and TGR5-Akt-NF-κB modulation

The gut microbiota is a central regulator of host metabolic networks and systemic health. Our metagenomic analysis revealed that CLP supplementation significantly increased the Shannon index and reshaped the microbial community structure. Such an increase in microbial complexity is characteristic of a more resilient and mature gut ecosystem, which is better equipped to withstand the perturbations associated with weaning stress [7]. This observation aligns with the prebiotic effects of bioactives from raspberry and honeysuckle, which selectively foster a more robust and antiinflammatory microbial state to facilitate colonic recovery [47, 48]. By providing a unique carbon source for beneficial fiber-fermenting taxa such as *Blautia* sp., *Eubacterium* sp., and *Ruminococcus* sp., CLP facilitates the colonization of obligate anaerobes specialized in degrading complex polysaccharides [30, 41]. Similar protective mechanisms have been reported for alfalfa and licorice derived polysaccharides, which promote the colonization of beneficial microbes to suppress pathobiont-induced inflammation [49, 50].

Intriguingly, we observed a significant contraction in the relative abundance of *Lactobacillus johnsonii* in the CLP group, a species that was overwhelmingly dominant in the control group. While *Lactobacillus* species are generally regarded as beneficial, their dominance is often characteristic of the suckling phase due to their specialization in lactate metabolism. The shift observed here likely represents an accelerated ecological succession from an immature microbiota associated with lactation- to a more mature fiber-fermenting community capable of utilizing complex polysaccharides. This niche replacement allowed for the expansion of specialized fiber degrading taxa, such as *Blautia* sp. and *Eubacterium* sp., which are critical for the production of short chain fatty acids and SBAs. Therefore, the reduction in *Lactobacillus* induced by CLP should not be viewed as a loss of beneficial microbes, but rather as an optimization of the gut ecosystem structure, enhancing its functional capacity to metabolize plant-based diets and produce anti-inflammatory metabolites like HDCA.

The reconstitution of the microbial assembly directly translated into clinical improvements and fortified barrier integrity. The significantly reduced relative abundance of *Escherichia coli*, a taxon frequently associated with intestinal dysbiosi*s,* in the CLP group was consistent with the reduced incidence of diarrhea and improved intestinal barrier function. This finding was further supported by the concomitant increase in goblet cell abundance and tight junction protein expression, specifically Occludin and ZO-1, which underscores the role of the CLP modified microbiota in preserving mucosal integrity. Such barrier enhancement recapitulates the protective effects reported for β-glucan and other prebiotics that resist pathogen colonization through competitive niche exclusion or the production of inhibitory metabolites [45, 51, 52]. Collectively, CLP promotes a shift from a dysbiotic state associated with weaning to a stable fiber-fermenting community. This ecological transition not only excludes pathobionts but also fortifies the mucosal barrier.

Beyond structural fortification of the mucosal barrier, the stabilization of the microbial community by CLP appears critical for metabolic homeostasis. Recent evidence suggests that prebiotics can restore the balance of bioactive metabolites by facilitating syntrophic metabolic consortia required for the multistep dehydroxylation of primary BAs into HDCA [18]. These BAs represent vital signaling links in the gut-liver axis, and porcine specific species like HDCA are gaining prominence for their unique therapeutic potential in metabolic and inflammatory diseases [53, 54]. Notably, HDCA producing species such as *Bifidobacterium pseudolongum* have been shown to attenuate colitis by generating bioactive metabolites that modulate host immune receptors[55, 56]. In the present study, FMT trials established a causal link between the remodeled microbiota and host protection by demonstrating that the amelioration of colonic injury was fundamentally dependent on microbiota-mediated metabolic activity. A hallmark of this microbial reconfiguration was the robust enrichment of HDCA, a phenomenon that aligns with findings where polysaccharides from *Acanthopanax senticosus* specifically promoted HDCA production to suppress proinflammatory responses [57]. These observations are further supported by evidence that maternal inulin supplementation can dramatically elevate porcine fecal HDCA concentrations by over 250%, highlighting the central role of microbiota-driven BA metabolism in shaping intestinal immune and metabolic homeostasis.[11].

Mechanistic evaluation identified the TGR5-Akt-NF-κB axis as the predominant circuit driving this protective effect. Although both TGR5 and FXR were assessed as potential targets, RT-qPCR analysis revealed that *Tgr5* mRNA levels exhibited a far more substantial increase compared to those of *Fxr*. This disparity was even more evident at the protein level, where Western blot analysis showed no significant change in FXR abundance in contrast to the robust upregulation of TGR5. These experimental results were strongly supported by molecular docking simulations, which demonstrated that HDCA possesses a superior binding affinity for the membrane bound TGR5 receptor over the nuclear receptor FXR, with binding energy scores of −8.8 kcal/mol and −6.8 kcal/mol respectively. Similar selective receptor activation has been documented in other metabolic contexts where HDCA acts as a high affinity ligand for TGR5 to trigger downstream protective responses [18, 19].

Given that TGR5 activation is a critical step in initiating downstream cascades that preserve epithelial integrity, the suppression of the PI3K-Akt-NF-κB signaling pathway by HDCA-mediated activation of TGR5 appears to be the primary mechanism for restoring colonic homeostasis. This observation mirrors broader findings whereby inhibiting the Akt-NF-κB signaling axis remodels the inflammatory microenvironment and thereby preventing excessive proinflammatory cytokine release across various disease models [20, 58, 59]. Specifically, recent research has demonstrated that TGR5 activation can effectively inhibit the phosphorylation of Akt and the subsequent nuclear translocation of NF-κB, thereby alleviating inflammatory damage in both intestinal and extra-intestinal tissues [24, 60]. By orchestrating this signaling framework, CLP derived HDCA provides a sophisticated defense against the epithelial damage and inflammation typically associated with weaning stress, ensuring both immediate signaling responses through the TGR5-Akt pathway and the maintenance of long-term homeostatic control.

The activation of the TGR5-Akt signaling axis by CLP-derived HDCA culminated in the profound suppression of the NF-κB signaling pathway, as evidenced by the diminished phosphorylation of the p65 subunit and IκBα. Mechanistically, the phosphorylation of IκBα at the Ser32/36 residues is a pivotal early event in the canonical activation of this pathway, which triggers its subsequent ubiquitination and proteasomal degradation [61]. By stabilizing IκBα and decreasing its phosphorylation, CLP intervention effectively prevents the liberation of NF-κB dimers, thereby retaining them in an inactive state within the cytoplasm. Furthermore, the phosphorylation of p65 at Ser536 is critical for transactivation activity and nuclear translocation, and its reduction confirms the pathway inactivation [62]. This provides further confirmation that CLP effectively suppresses the inflammatory cascade. This molecular braking of the NF-κB cascade represents a convergent mechanistic hallmark shared among several highly efficient bioactive polysaccharides. For instance, the synbiotic combination of galactooligosaccharides and *Limosilactobacillus reuteri* has been shown to synergistically inhibit NF-κB activation by promoting the biosynthesis of pentadecanoic acid [31]. Similarly, the capacity of CLP to abrogate the p65 and IκBα phosphorylation cascade recapitulates the therapeutic effects reported for *Chrysophyta* polysaccharide, pectin polysaccharides, and *Pleurotus citrinopileatus* polysaccharides, all of which have been demonstrated to effectively dampen the secretion of proinflammatory cytokines such as IL-1β and TNF-α [48, 63, 64]. Furthermore, the capacity of CLP to mitigate neutrophil infiltration, which is evidenced by reduced MPO activity, parallels the antiinflammatory efficacy of other prebiotics that target the TLR4/NF-κB axis [65]. The specificity of CLP in blocking NF-κB translocation mirrors the therapeutic action of black mulberry and almond polysaccharides [66], as well as raspberry pectin [48], all of which converge on the abrogation of the p65 phosphorylation cascade to restore mucosal homeostasis.

## Conclusions

In conclusion, dietary supplementation of CLP effectively alleviates weaning stress and colonic inflammation in piglets. These beneficial effects are associated with a reshaped gut microbiota and increased HDCA production, which subsequently activate the colonic TGR5-Akt-NF-κB signaling pathway as validated in PGF mouse and in vitro models. Our findings provide evidence supporting the potential of CLP as a functional feed additive for promoting intestinal health during the weaning transition.

## Supplementary Information

Additional file 1: Table S1. Scoring system for histological colonic damage. Table S2. Scoring criteria for the disease activity index in mice. Table S3. Information on the key ELISA kits used in the experiments. Table S4. The primary antibody information of Immunofluorescence analysis. Table S5. Secondary antibodies and detection reagents used for immunofluorescence analysis. Table S6. Primer sequences used in the present study. Table S7. Primary antibodies information of Western blot analysis.

Additional file 2. Full uncropped blots images.

## Data Availability

The data used to support the findings of this study are available from the corresponding authors upon request.

## Abbreviations

AB-PAS: Alcian blue-periodic acid-Schiff
ABX: Antibiotic
ADF: Acid detergent fiber
ADG: Average daily gain
ADFI: Average daily feed intake
ATTD: Apparent total tract digestibility
BAs: Bile acids
BW: Body weight
CLP: *Cichorium intybus* L. polysaccharide
CP: Crude protein
DAI: Disease activity index
DM: Dry matter
DSS: Dextran sulfate sodium
ELISA: Enzyme linked immunosorbent assay
F:G: Feed to gain ratio
FMT: Fecal microbiota transplantation
GE: Gross energy
H&E: Hematoxylin and eosin
HDCA: Hyodeoxycholic acid
HI: Histology index
HRP: Horseradish peroxidase
IL-1β: Interleukin-1β
IL-6: Interleukin-6
IL-10: Interleukin-10
MFI: Mean fluorescence intensity
MPO: Myeloperoxidase
NDF: Neutral detergent fiber
OM: Organic matter
PGF: Pseudo-germ-free
RT-qPCR: Reverse transcription quantitative PCR
SBAs: Secondary bile acids
TNF-α: Tumor necrosis factor-α
TSA: Tyramide signal amplification
WB: Western blotting
