# Supplementary material for: Cichorium intybus L. polysaccharide improves growth performance and colonic barrier function in weaned piglets via the microbiota-HDCA-TGR5-Akt-NF-κB signaling axis: validation by FMT and in vitro models
Source: J Anim Sci Biotechnol. 2026 Jul 6;17:140. doi: 10.1186/s40104-026-01449-0 (PMC13335359; doi:10.1186/s40104-026-01449-0)
Supplement: Supplementary file 1 — Additional file 1: Table S1. Scoring system for histological colonic damage. Table S2. Scoring criteria for the disease activity index in mice. Table S3. Information on the key ELISA kits used in the experiments. Table S4. The primary antibody information of Immunofluorescence analysis. Table S5. Secondary antibodies and detection reagents used for immunofluorescence analysis. Table S6. Primer sequences used in the present study. Table S7. Primary antibodies information of Western blot analysis. [file 40104_2026_1449_MOESM1_ESM.docx]

Table S1 Scoring system for histological colonic damage

| **Epithelial damage** | **Inflammation severity** | **Lesion depth** | **Score** |
| --- | --- | --- | --- |
| Intact mucosa | None | None | 0 |
| Irregular crypts and goblet cell loss | Minimal | Crypt | 1 |
| Mild to moderate crypt loss | Mild | Lamina propria | 2 |
| Severe crypt loss | Moderate | Submucosa | 3 |
| Total crypt and epithelial destruction | Severe | Muscularis/Serosa | 4 |

**Note:** Histology index (HI) = epithelial damage score + inflammation severity score + lesion depth score

Table S2 Scoring criteria for the disease activity index in mice

| **Weight loss** | **Stool consistency** | **Fecal blood** | **Score** |
| --- | --- | --- | --- |
| < 1% | Normal | Negative | 0 |
| 1-6% | Soft but formed | Brownish stool | 1 |
| 6-12% | Loose stools | Slightly reddish | 2 |
| 12-18% | Very soft and moist | Gross bleeding | 3 |
| > 18% | Watery diarrhea | Rectal bleeding | 4 |

**Note:** DAI = (weight loss score + stool consistency score + fecal blood score) / 3.

Table S3 Information on the key ELISA kits used in the experiments

| **Items** | **Species** | **Intra-assay CV** | **Inter-assay CV** | **Sensitivity** | **Detection Range** | **Cat. No.** | **Company** |
| --- | --- | --- | --- | --- | --- | --- | --- |
| IL-1β | Pig | < 10% | < 15% | 2.0 ng/L | 25-800 ng/L | ELS875 | Beijing Laiboterui Technology Development Co., Ltd., Beijing, China |
|  | Mouse | < 10% | < 15% | 0.5 pg/ml | 18.75-600 pg/ml | ELS295 |  |
| IL-6 | Pig | < 10% | < 15% | 2.0 ng/L | 10-320 ng/L | ELS879 |  |
|  | Mouse | < 10% | < 15% | 1.0 pg/ml | 18.75-600 pg/ml | ELS300 |  |
| IL-10 | Pig | < 10% | < 15% | 1.0 ng/L | 5-160 ng/L | ELS882 |  |
|  | Mouse | < 10% | < 15% | 0.5 pg/ml | 6.25-200 pg/ml | ELS303 |  |
| TNF-a | Pig | < 10% | < 15% | 1.0 ng/L | 15-480 ng/L | ELS889 |  |
|  | Mouse | < 10% | < 15% | 2.0 pg/ml | 100-3200 pg/ml | ELS286 |  |
| MPO | Pig | < 10% | < 15% | 2.0 U/L | 50-800 U/L | ELS959 |  |
|  | Mouse | < 10% | < 15% | 1.0 U/L | 5-240 U/L | ELS309 |  |
| Total Protein (TP) | Pig / Mouse | ≤ 2% | ≤ 5% | 20 mμg/mL | 20-2000 | A045-3 | Nanjing Jiancheng Bioengineering Institute, Nanjing, China |

Table S4 The primary antibody information of immunofluorescence analysis

| **Antibody name** | **Company** | **Host Species** | **Cat. No.** | **Dilution rate** |
| --- | --- | --- | --- | --- |
| Occludin | Servicebio, Beijing, China | Mouse | GB15149 | 1:2000 |
| MUC2 | Servicebio, Beijing, China | Mouse | GB120002 | 1:5000 |
| ZO-1 | Servicebio, Beijing, China | Mouse | GB12195 | 1:2000 |

Table S5 Secondary antibodies and detection reagents used for immunofluorescence analysis

| **Reagent name** | **Company** | **Application / Spectrum** | **Cat. No.** | **Dilution rate** |
| --- | --- | --- | --- | --- |
| HRP-conjugated Goat Anti-Mouse IgG | Servicebio, Beijing, China | Secondary Antibody | G1301 | Ready-to-use |
| iF488-Tyramide | Servicebio, Beijing, China | Ex/Em: 491/516 nm | G1231 | 1:500 |
| iF555-Tyramide | Servicebio, Beijing, China | Ex/Em: 557/570 nm | G1233 | 1:500 |
| iF647-Tyramide | Servicebio, Beijing, China | Ex/Em: 656/670 nm | G1232 | 1:500 |
| DAPI | Servicebio, Beijing, China | Ex/Em: 359/457 nm | G1012 | Ready-to-use |

**Table S6** **Primer sequences used in the present study**

| **Genes** | **Forward (5’-3’)** | **Reverse (5’-3’)** | **Product size (bp)** | **Accession number** |
| --- | --- | --- | --- | --- |
| Mus *Fxr* | CTTCGTTCGGCGGAGATT | CACTTTTATAGAAACTGAACATCGG | 127 | NM_001163700.1 |
| Human *Fxr* | GACTTTGGACCATGAAGACCAG | GCCCAGACGGAAGTTTCTTATT | 104 | NM_001206977.2 |
| Mus *Tgr5* | TCCTGTCAGTCTTGGCCTATGA | GGTGCTGCCCAATGAGATG | 80 | NM_174985.2 |
| Human *Tgr5* | CCTGCTCCCAACAGCCATCT | AGCCTCCGGGTAGGACTTCC | 95 | NM_001077191.2 |
| Mus *GAPDH* | GGTTGTCTCCTGCGACTTCA | TGGTCCAGGGTTTCTTACTCC | 183 | NM_001411840.1 |
| Human *GAPDH* | CATGGCAAATTCCATGGCAC | GATTTTGGAGGGATCTCGCT | 99 | NM_001289745.3 |

**Table S7** **Primary antibodies information of western blot analysis**

| **Antibody** | **Cat. No.** | **Concentration** | **Company** |
| --- | --- | --- | --- |
| FXR | M022312 | WB: 1:1500 | Abmart Shanghai Co.,Ltd., Shanghai, China |
|  |  |  |  |
| TGR5 | ab72608 | WB: 1:1000 | Abcam Public Limited Company Cambridge, UK |
|  |  |  |  |
| Akt | T55561 | WB: 1:1000 | Abmart Shanghai Co.,Ltd., Shanghai, China |
| p-Akt (Ser473) | T40067 | WB: 1:1000 | Abmart Shanghai Co.,Ltd., Shanghai, China |
| p65 | T55034 | WB: 1:5000 | Abmart Shanghai Co.,Ltd., Shanghai, China |
|  |  |  |  |
| p-p65 | TP56372 | WB: 1:1000 | Abmart Shanghai Co.,Ltd., Shanghai, China |
|  |  |  |  |
| IKBα | T55026 | WB: 1:1000 | Abmart Shanghai Co.,Ltd., Shanghai, China |
|  |  |  |  |
| p-IKBα | 82349-1-RR | WB: 1:2000 | Proteintech Group, Inc, Wuhan, China |
|  |  |  |  |
| β-actin | 66009-1-Ig | WB: 1:20000 | Proteintech Group, Inc, Wuhan, China |
