# Supplementary figures and images for: Cichorium intybus L. polysaccharide improves growth performance and colonic barrier function in weaned piglets via the microbiota-HDCA-TGR5-Akt-NF-κB signaling axis: validation by FMT and in vitro models
Source: J Anim Sci Biotechnol. 2026 Jul 6;17:140. doi: 10.1186/s40104-026-01449-0 (PMC13335359; doi:10.1186/s40104-026-01449-0)

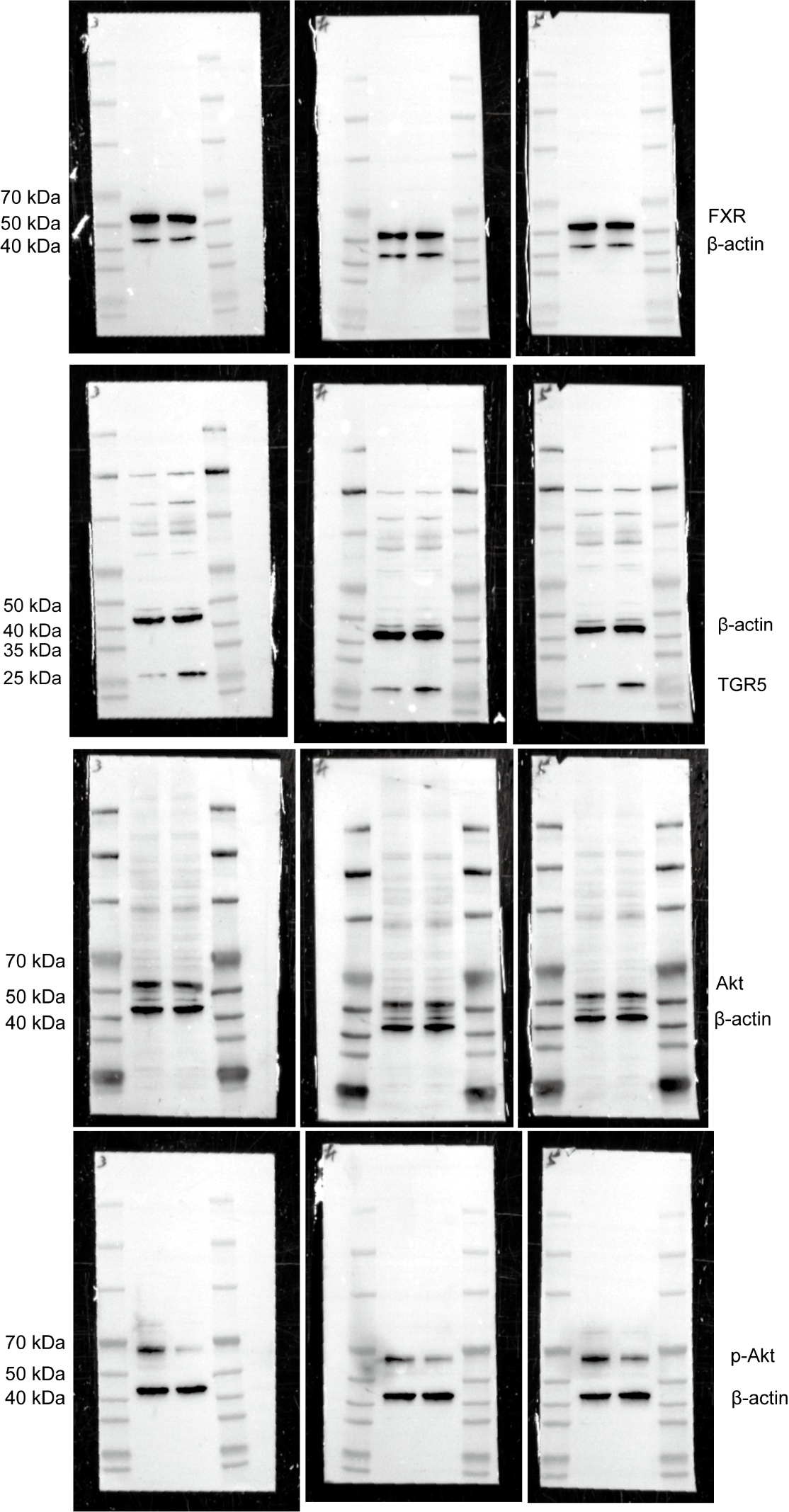


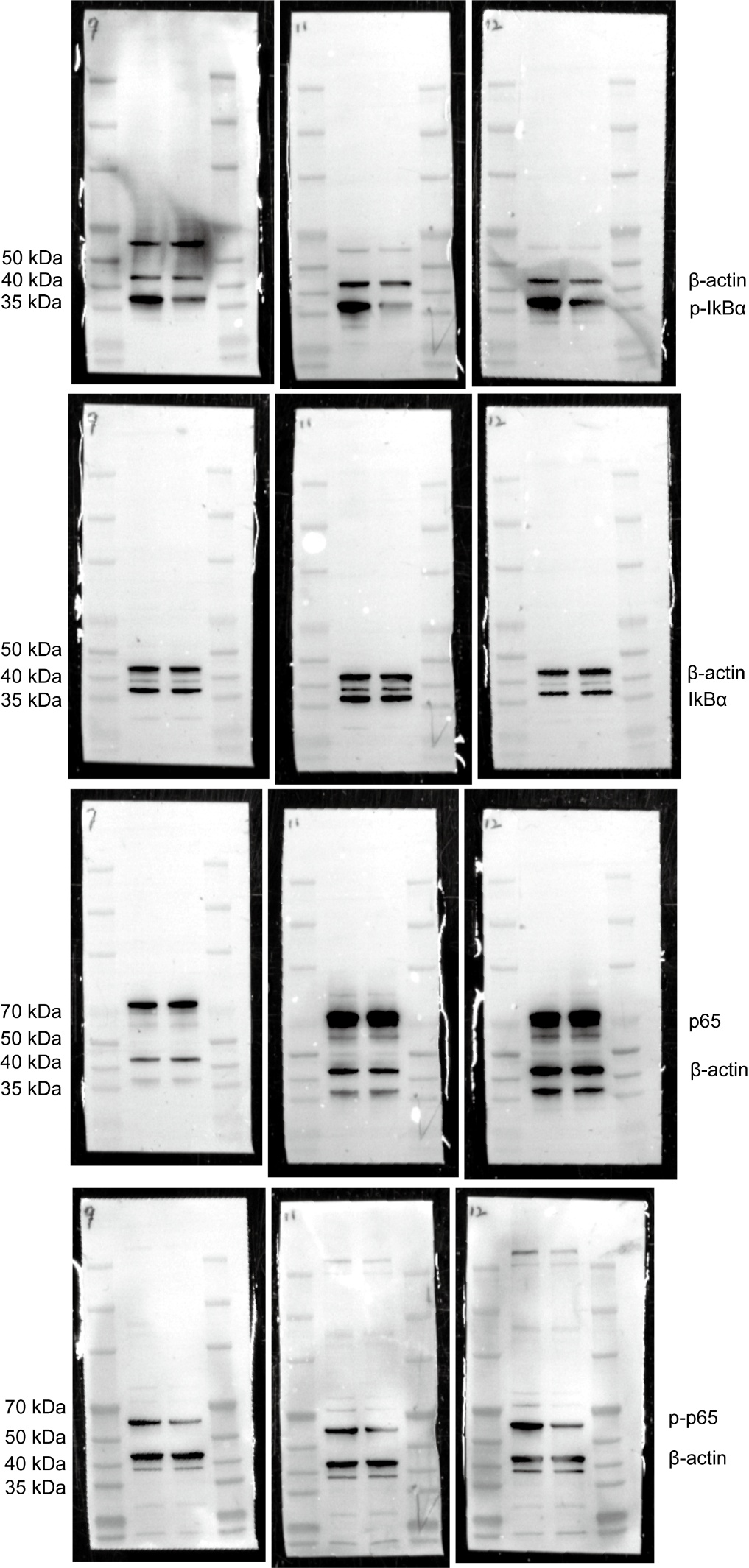

Supplement: Supplementary file 2 — Additional file 2. Full uncropped blots images. [file 40104_2026_1449_MOESM2_ESM.docx]
